# TAP score: torsion angle propensity normalization applied to local protein structure evaluation

**DOI:** 10.1186/1471-2105-8-155

**Published:** 2007-05-15

**Authors:** Silvio CE Tosatto, Roberto Battistutta

**Affiliations:** 1 Dept. of Biology and CRIBI Biotechnology Centre, University of Padova, Italy; 2 Dept. of Chemical Sciences and Venetian Institute of Molecular Medicine (VIMM), University of Padova, Italy

## Abstract

**Background:**

Experimentally determined protein structures may contain errors and require validation. Conformational criteria based on the Ramachandran plot are mainly used to distinguish between distorted and adequately refined models. While the readily available criteria are sufficient to detect totally wrong structures, establishing the more subtle differences between plausible structures remains more challenging.

**Results:**

A new criterion, called TAP score, measuring local sequence to structure fitness based on torsion angle propensities normalized against the global minimum and maximum is introduced. It is shown to be more accurate than previous methods at estimating the validity of a protein model in terms of commonly used experimental quality parameters on two test sets representing the full PDB database and a subset of obsolete PDB structures. Highly selective TAP thresholds are derived to recognize over 90% of the top experimental structures in the absence of experimental information. Both a web server and an executable version of the TAP score are available at .

**Conclusion:**

A novel procedure for energy normalization (TAP) has significantly improved the possibility to recognize the best experimental structures. It will allow the user to more reliably isolate problematic structures in the context of automated experimental structure determination.

## Background

The number of experimentally determined protein three-dimensional (3D) structures deposited in the protein data bank (PDB) [1] is increasing exponentially over the years and being progressively automated. The vast majority of such 3D structures is produced by X-ray crystallography. In the case of limited resolution and imperfect phase information often available to the crystallographer, building and refining such a protein model is a process that can depend on the experimentalist. Errors are almost unavoidable and the quality of the refined models has to be evaluated in order to assess their validity [2]. Errors come in various classes [3] and can nowadays range from mistraced segments of the protein to locally incorrect backbone and/or side chain conformations. Identification of such errors can be achieved with a combination of experimental and computational parameters.

Several quality measures based on experimental parameters exist for X-ray crystallography and have been reviewed [2]. X-ray resolution is an index of the quality of the experimental data. It is related to the amount of available data, i.e. to the parameter-to-observation ratio [4] and is an indirect indicator of the maximum attainable details of the protein model. In contrast, the R-free value [5] represents a measure of the fit between the refined structure and the electron density map, highlighting the quality of the refinement. The relationship with resolution is not straightforward, but it is generally assumed that higher resolution structures will produce lower R-free values [6]. A different type of information is contained in the Luzzati [7] and σ_a _[8] plots. These estimate the mean positional error for all atoms in the protein model. These parameters require the structure factors, but yield the expected uncertainty of atoms in the protein model in a single number estimate (in Å). A simpler estimation of the mean positional error is the diffraction precision index based on R-free (DPI), which can be computed from the readily available data contained in the PDB files [9].

A wide range of computational quality parameters have been developed and reviewed over the years [2,10,11]. Generally speaking, it is possible to distinguish geometric, energetic and conformational criteria. Geometric criteria are mainly standard values for bond lengths and angles derived from small molecule data. These form strong restraints and are generally enforced during the refinement process, so they possess little validation power. Energetic criteria are based on evaluation of interaction preferences or profiles [12-16]. While these methods can provide insight into the quality of the structure, their interpretation in experimental terms and feedback into the refinement process is rather difficult.

The most promising validation criteria are based on conformational criteria. The best example is the Ramachandran plot [17] of backbone (ϕ,φ) torsion angles. While each amino acid type may, in theory, adopt a large number of different conformations, large areas of the Ramachandran plot are almost empty. This is due to steric clashes deriving from the local geometry of the polypeptide chain. The main chain (ϕ,φ) torsion angles are usually not restrained during refinement and this makes the Ramachandran plot a powerful validation tool [2,18]. Several tools have been developed to estimate the quality of a protein model based on the Ramachandran plot [16,18-22]. Of these, PROCHECK [19] and WHAT_CHECK [16] are perhaps the most frequently used methods for validation in X-ray crystallography as they are used for judging structures to be deposited in the PDB, combining several stereochemical checks and measures of torsion angle compatibility. HOPPscore [22] has been recently developed to take into account higher order backbone torsion angle maps.

Several of these methods (e.g. WHAT_CHECK) are able to pinpoint the really wrong structures through a detailed analysis of different aspects of protein structures. Once a structure falls into the range of roughly plausible folds however the situation becomes more complicated. It is possible to construct structures with acceptable values for the standard criteria that are largely incompatible with the protein sequence. In the present work we focus on this aspect of experimental structure validation. Given roughly plausible structures, is it possible to quantify the degree of "nativeness" and highlight the best structures?

One possible limit to the previous methods is the difficulty in establishing a quantitative correspondence scale between different structures. I.e. how much is score X for structure A better or worse than score Y for structure B? The answer is not obvious, as the reference state is different for structures A and B. One solution would be a normalization procedure adapted to a particular structure. To the best of our knowledge, this has not been done yet. For this reason, we derive a novel measure of sequence to structure compatibility based on the normalization of torsion angle propensities including the side chain. The normalization involves definition of the global minimum and maximum of the protein sequence. The normalized propensity (called TAP) will be shown to be more accurate than several previous methods at quantifying the degree of "nativeness" of a protein model in terms of commonly used experimental quality parameters on two test sets representing the full PDB database and obsolete PDB structures. A comparison with several energetic criteria on standard protein decoys and theoretical models has already been addressed elsewhere [23,24].

## Results

### Baseline comparison on the all PDB set

Available experimentally derived quality parameters for the all PDB set representing 13,691 structures is summarized in Table 1. While the X-ray resolution information is available for all structures considered, some of the older structures are lacking an R-free value. The cross-validated Luzzati and σ_A _plots are present only in roughly one out of three structures, mainly because deposition was not mandatory for a long time. While it is possible to calculate both parameters from the structure factors, we have chosen to limit our comparison only to the publicly available cases.

**Table 1:** Availability of data for the all PDB set.

	**Structures**	**SCOP families**
**Resolution**	17,330	2,502
**R-free**	14,208	2,435
**σ_A_**	5,373	1,536
**Luzzati**	5,564	1,586
**DPI**	16,726	2,493
**All PDB set**	13,691	2,435

**Figure 1:**
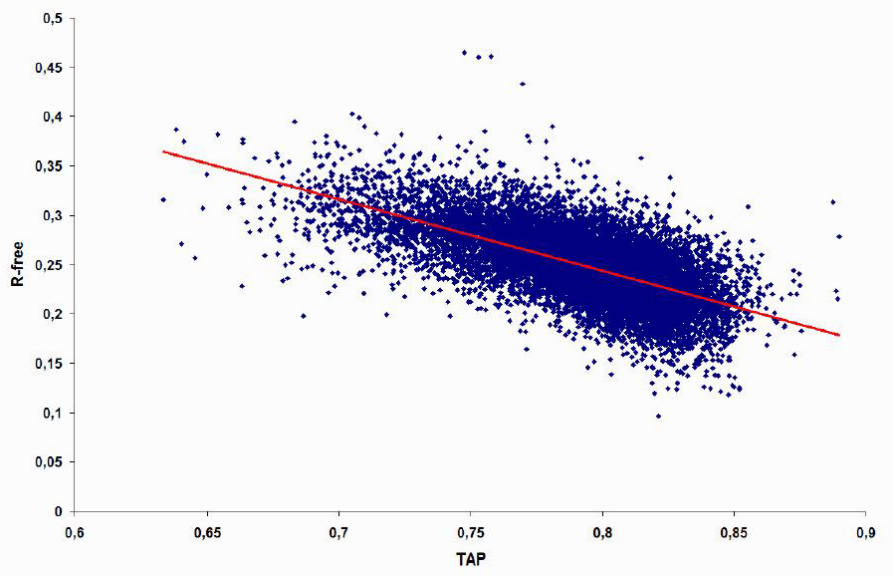
**TAP-10 vs. R-free scatter plot on the all PDB set**. The distribution of TAP score vs. R-free is shown for 13,691 structures in the all PDB set, together with the corresponding linear regression (red line). The correlation coefficient is -0.652.

The Pearson correlation coefficients (cc) between the different experimental quality parameters were calculated for the available data (see Table 2). As expected, the correlation between experimental parameters is usually very high (cc > 0.8). The main exception is R-free with cc <= 0.62 to the X-ray resolution and DPI (see also Figure 1). As information contained in the various measures appears largely redundant, we restrict further analysis to resolution and R-free. R-free is not a perfect measure, but rather available for more structures and, perhaps, less inaccurate than the other quality parameters.

**Table 2:** Correlation coefficients between different experimental quality parameters on the all PDB set.

	**R-free**	**σ_A_**	**Luzzati**	**DPI**
**Resolution**	0.62	0.80	0.86	0.86
**R-free**		0.66	0.82	0.55
**σ**_A_			0.87	0.77
**Luzzati**				0.84

In order to estimate the performance of available computational methods, we compare our method with PROCHECK [19], WHAT_CHECK [16], HOPPscore [22] and FRST [23]. This covers all the range from energetic to geometric and conformational criteria. Table 3 shows the Pearson correlation coefficients between computational and experimental parameters for the all PDB set. The best performance is seen for methods using Ramachandran plot analysis, i.e. TAP and WC_Rama, once again confirming its utility in structure validation [2,10].

**Table 3:** Correlation coefficients for the computational parameters on the all PDB set.

	**Resolution**	**R-free**
**TAP**	-0.720	-0.652
**TAP-20**	-0.693	-0.640
**TAP-5**	-0.678	-0.609
**TAP-NMR**	-0.557	-0.530
**PROCHECK**	-0.092	-0.135
**FRST**	0.503	0.360
**RAPDF**	0.069	-0.060
**SOLV**	0.054	-0.030
**HYDB**	-0.044	-0.001
**TORS**	0.550	0.476
**WC_Qual**	-0.524	-0.441
**WC_Tors**	-0.373	-0.343
**WC_Rama**	-0.714	-0.596
**WC_Chir**	-0.073	-0.108
**WC_Back**	-0.135	-0.113
**WC_Rot**	-0.104	-0.134
**WC_Chi1&2**	-0.613	-0.567
**WC_Pack1**	-0.295	-0.257
**WC_Pack2**	-0.331	-0.281
**Hopp5**	-0.539	-0.410
**Hopp4**	-0.467	-0.361
**Hopp3**	-0.523	-0.421
**Hopp2**	-0.602	-0.503
**Hopp1**	-0.586	-0.478

For the TAP score, the intermediate (ϕ,φ) bin size of 10 degrees shows the highest correlation (see also Figure 1). A similar trend was already observed [24]. This probably maximizes the tradeoff between precise transitions and lack of data to discriminate certain sparsely populated regions in the Ramachandran plot. Note that a larger background distribution, e.g. covering the entire PDB, was excluded to avoid biasing the comparison.

It is apparent from Table 3 that some methods work significantly better than others. The statistical potentials (except the Ramachandran plot based TORS) and several geometric criteria do not yield good correlation coefficients. Performance of the TAP score against R-free is particularly interesting, as the correlation coefficients for R-free are overall lower. TAP has a higher correlation against R-free (-0.66; see Table 3) than the X-ray resolution (0.62; see Table 2) has. In order to evaluate the effect of the background distribution on the performance of TAP, a further test was made using the TAP score based on NMR derived torsion angles. The data reported in Table 3 shows that, while the usage of high-quality data improves the performance, TAP-NMR still significantly outperforms many other methods. For the sake of simplicity, further analysis was restricted to TAP and the most diverse parameters. As conformational criteria we have chosen PROCHECK, WC_Rama, WC_Chi1&2, Hopp1, Hopp2 and Hopp5. For the energetic criteria we have restricted our analysis to WC_Pack2, SOLV, RAPDF and TORS.

### Detailed comparison on the obsolete PDB set

**Figure 2:**
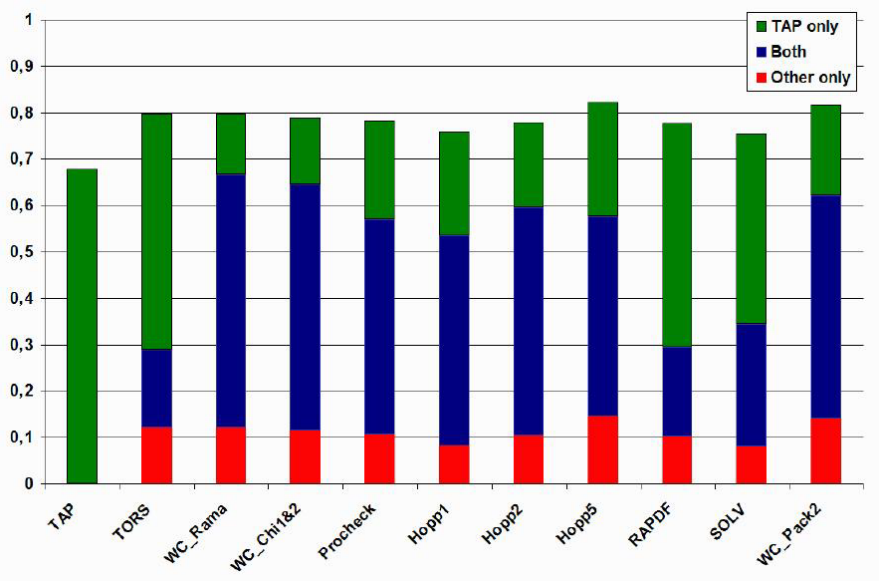
**Histogram of correctly recognized improved structures on the obsolete PDB set for TAP and ten previous methods**. The number of correct predictions by both methods (blue), TAP only (green) and only the second method (red). The total height corresponds to the performance of combining TAP and the other method. Individual performance of each method (except TAP) is the sum of the first two terms (i.e. red and blue).

**Figure 3:**
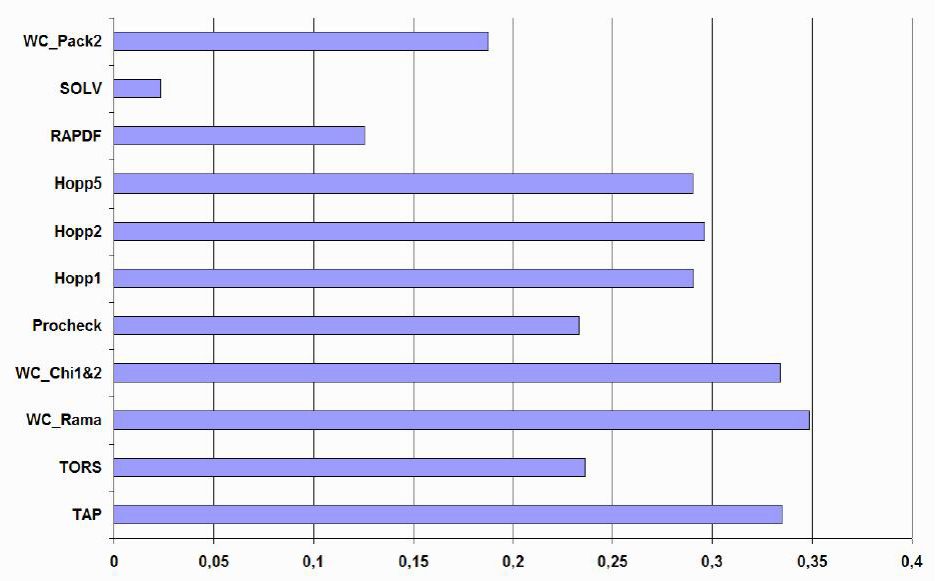
Histogram of separation Z-score on the obsolete PDB set for TAP and ten previous methods.

A set of 494 pairs of obsolete PDB structures and their replacement was analyzed in order to evaluate the capacity of the TAP score to discriminate better models for the same protein. Figure 2 shows the number of times each method correctly assigned a better score to the newer, and therefore more accurate, model in the obsolete PDB set. The results for each individual methods and the combination with TAP are shown. None of the methods discriminates all 494 structures, and the statistical potentials in particular have difficulty recognizing the improved structures, while TAP has one of the highest single recognition rates. Combining methods improves the overall performance, with TAP typically contributing more unique information than the other method. Figure 3 shows a different analysis of the data in terms of separation Z-score, i.e. the normalized difference between the obsolete and replacement structure. Here it is again apparent that the Ramachandran plot based methods outperform the others, with TAP coming a close second with WC_Chi1&2 after WC_Rama. Taken together, the Ramachandran plot based methods (especially TAP and WC_Rama) seem able to qualitatively discriminate improved from obsolete structures.

### Quantifying absolute model accuracy

**Figure 4:**
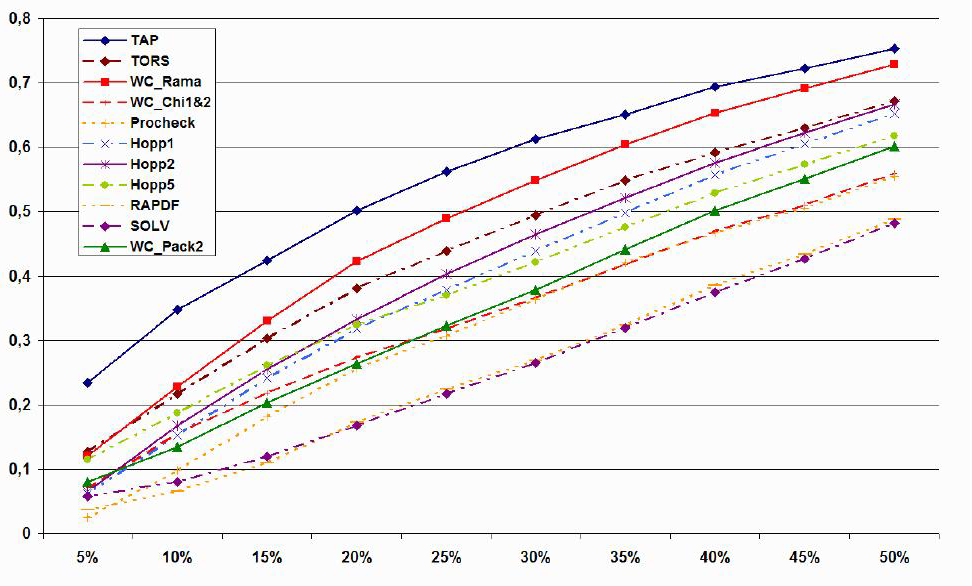
Fraction enrichment plot for TAP and ten previous methods on the all PDB set.

Another interesting question related to structure quality is whether the methods under consideration are able to quantify the difference between structures, i.e. reliably identify the top x% structures present in the PDB. This problem was addressed in terms of fraction enrichment on R-free (see Materials and Methods), with results shown in Figure 4. Unsurprisingly, the statistical potentials perform worse than the conformational methods. Of the latter group, it is worth noting how WC_Chi1&2, based on side chain rotamer preferences, performs worse than methods using backbone preferences. The difference between TAP and WC_Rama suggests that energy normalization is improving recognition especially with the highest quality structures. This idea is confirmed when comparing TAP to TORS, the torsion angle propensity energy before normalization.

### Confidence estimates

Since it can be very useful to derive TAP score threshold levels indicating the expected quality of a model, the all PDB set was used to derive confidence estimates. The resolution and R-free parameter distributions were analyzed on the all PDB set to estimate average and standard deviation (σ) (see Table 4). Three derived threshold levels are shown in Table 5. The medium quality class is based on commonly accepted conservative parameter settings, i.e. resolution <= 2.5 A and R-free < 0.30, and excludes the 25% lowest quality structures. The high quality class was defined to be just above the average for resolution and R-free, selecting ca. the 40% top structures. The very high quality class was defined at one σ above average and represents ca. the 10% top structures. The distribution therefore appears not to be truly normal, but somewhat skewed towards lower quality structures. Similarly, TAP score thresholds were chosen from the average to be – 1 σ (low), 0 (medium) and + 1 σ (high).

**Table 4:** Distribution of parameters for the all PDB set. Minimum, maximum, average and standard deviation are shown.

	**Resolution**	**R-free**	**TAP**
**Min**	0.88	0.096	0.633
**Max**	6.21	0.465	0.890
**Avg**	2.17	0.249	0.792
**SD**	0.46	0.036	0.032

**Table 5:** Threshold levels used to define the experimental quality classes on the all PDB set.

	**Medium**	**High**	**Very High**
**Resolution**	<= 2.5	<= 2.2	<= 1.7
**R-free**	<= 0.3	<= 0.25	<= 0.22
**Relative frequency**	76.7%	42.2%	8.7%
**# Cases**	10,771	5,926	1,222

The results for TAP on all three experimental quality classes are expressed in terms of accuracy and coverage (see Materials and Methods) and shown in Table 5 for the all PDB set. As can be expected, it becomes gradually more difficult for TAP to discriminate the structures with increasing quality level. At the same time, coverage drops with increasing TAP threshold. Taking the intersection between both, TAP recognizes ca. 90% of the medium quality structures with 90% accuracy. These values drop to ca. 75% for the high and 35% for the very high quality structures. Even in the latter case it implies a significant enrichment in discrimination with respect to a random predictor. To the best of our knowledge, this type of analysis was not performed before. In the context of automated experimental structure determination, it will allow the user to isolate problematic structures for manual refinement and could prove a valid addition to the PDB data deposition procedures.

## Discussion

### Database effects vs. novel approach

The Ramachandran plot, i.e. (ϕ,φ) torsion angle preferences, has been seen as a powerful tool for validating experimental protein models for a long time [10,18-20,22]. Usually, the Ramachandran plot is used only as a rather qualitative tool to discriminate grossly mistraced structures from plausible ones. PROCHECK and HOPPscore both consider a generic Ramachandran plot for the twenty amino acids divided in discrete classes. WHAT_CHECK-2 uses a more sophisticated Z-score analysis of the Ramachandran plot. All of these do not appear to discriminate effectively the compatibility between sequence and structure, nor the subtle differences between amino acids.

The main advantage of TAP consists in effectively measuring the compatibility of the sequence with the proposed structure in a detailed, quantitative way. Energy score normalization is a novel concept which could be applied because TAP is based on a single body potential. This is not usually applicable to pair wise (or higher order) potentials, where it is difficult to estimate the maximum or minimum interaction between an amino acid and its surroundings. The benefits of energy normalization are apparent from the comparison between TAP and TORS, the torsion angle potential on which it is based. Where TORS gives rough indications, TAP (despite using similar information) has greater accuracy.

Since torsion angles are not generally restrained in X-ray crystallography, this compatibility is orthogonal to the data used in refinement and should be expected to give a good indication of the degree of "nativeness" of the protein model. To support this view, rather than a simple improvement based on database growth, a variant of TAP was derived from NMR data. Even in this case, where the Ramachandran plot is on average rather blurred, TAP-NMR still outperforms other validation tools. This supports the idea that it is capturing the relationship between sequence and structure rather than a tighter clustering in torsion angle space.

**Figure 6:**
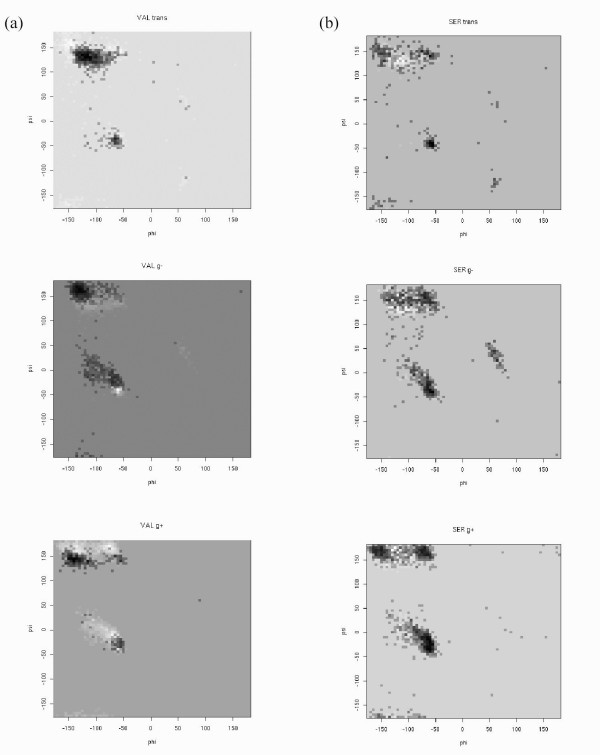
**Ramachandran plot of valine (a) and serine (b) residues**. In each plot, the χ1 torsion angle is fixed at a rotamer position (trans, g-, g+) and the (φ,ϕ) TAP score landscape (resolution 5° × 5°) is plotted from white to black. Darker colours represent higher (i.e. better) TAP scores. Note that the less favoured regions are those where a given residue is less frequently present than the average of the twenty types.

Perhaps the most important feature of the TAP score is that it simultaneously combines five different torsion angles into a single pseudo-energy value. Adding more torsion angles was previously shown to improve the overall discrimination of protein decoys [24], as it captures the subtle interplay between them. For instance, it is known that the ω torsion angle varies slightly depending on the (ϕ,φ) angles [10,25]. Even more widely used is the dependence of the χ torsion angles on (ϕ,φ), which is widely accepted in side chain modeling [26,27]. A TAP variant based solely on the (ϕ,φ) angles performs significantly worse, with correlation coefficients of -0.53 and -0.42 for resolution resp. R-free. This view has been recently reinforced by an elegant statistical and conformational analysis of the electron density of protein side chains showing a vast majority of all residues in high resolution X-ray structures to have rotameric side chain positions [28]. Therefore, where HOPPscore successfully extends the concept of Ramachandran plot to higher order (ϕ,φ) torsion angle pairs, TAP score explores the avenue of capturing the interplay between protein backbone and side chain. Figure 6 shows an example of TAP score depending on χ_1 _side chain conformation.

The main limit of the TAP score approach is the independence of subsequent residues in the calculation of the global minimum and maximum for normalization. These minimum and maximum are likely to be overestimated, as compatibility along the polypeptide chain is not guaranteed. This may result in impossibly "knotted" structures having the best pseudo-energy and the native structure being lower in normalized score. In principle, adding information about the preceding residue's (ϕ,φ) torsion angles would alleviate this situation and has been shown to yield more discriminative pseudo-energies [24]. However, it is only possible to calculate the global optimum for normalization precisely because it is a single body potential. Adding a dependence on the preceding residue would transform it into a two-body potential, making the estimation of the global optimum problematic. Calculating the global optimum on such a two-body potential is an optimization problem in itself.

The discriminative power of torsion angle propensities has implications for the accuracy of empirical force fields such as AMBER [29] or CHARMM [30]. Torsion angle propensities derive from the subtle interactions between neighboring residues which cannot be captured very precisely by currently available physico-chemical models [31]. This knowledge is one of the reasons for the success of modern de novo folding methods based on assembly of short peptide fragments [31-34].

Small changes in the AMBER torsion angle parameter between param94 and param96 have drastic effects on the energy landscape [35]. It may be argued that addition of a Ramachandran plot propensity parameter could improve the capacity of a force field to capture the local geometric details more precisely. This approach is frequently selected in loop modelling [36-38] where it is important to reconcile the structural restraints with sequence preference. The method of Fiser and co-workers [38] in particular uses the CHARMM bonded potential augmented by a Ramachandran plot propensity term and statistical non-bonded potential to generate loop conformations by minimization. Adding a properly calibrated (ϕ,φ) torsion angle propensity term to a force field may therefore help to improve convergence in energy minimization for molecular mechanics simulations. The option to calculate such values for every single residue also opens up the interesting possibility to use the TAP score as a valuable tool during refinement of a crystallographic model, in addition to the already available geometric validation tools.

An interesting question is why some crystal structures exhibit higher TAP scores than others. A cursory analysis reveals that the highest scoring structures are short helical bundles, e.g. Hemoglobin. The lowest scoring structures are diverse and contain any combination of α-helices and β-sheets. Even in the TOP500H database used for deriving the propensities, the TAP scores vary between 0.781 and 0.907 (avg = 0.847; σ = 0.017). This is comparable to the TAP score of very high experimental quality structures varying between 0.754 and 0.889 (avg = 0.822; σ = 0.017). This may be caused by the rough approximation of the global optimum overestimating some folds more than others due to some intrinsic feature, e.g. higher contact order or lower degree of flexibility. A better way to calculate the global optimum would be needed to exclude this possibility. As local interactions appear to impose the selection of certain amino acids at each structural position in the fold [39], it will however be worth investigating whether proteins whose native structure lies closer to the global optimum could perhaps also have a better sequence to structure compatibility.

## Conclusion

We have presented a novel method for the evaluation of the quality of protein models determined by X-ray crystallography, demonstrated both on a large-scale dataset and a set of obsolete PDB structures. The TAP score is based on a relative pseudo-energy calculated simultaneously from the backbone and side chain torsion angle propensities, normalized against the global minimum and maximum for the protein sequence under consideration. Our results show a quantitative relationship between TAP score and the overall quality of experimental structures as expressed in terms of sequence to structure compatibility. TAP score can improve the confidence in quality validation of protein models derived from automated experimental procedures.

## Methods

### Torsion angle potential

The torsion angle potential was developed as part of the FRST scoring function [23] for model quality estimation which later has been extended [24]. It is a statistical potential [40] based on torsion angle propensities calculated in analogy to the one defined by Shortle [41]. If *x *describes a discrete torsion angle combination and *A *is a particular amino acid type, a propensity *P *can be defined as the fraction of two probabilities:

PA(i),x(i)=(NA,xNANxNtotal)
 MathType@MTEF@5@5@+=feaafiart1ev1aaatCvAUfKttLearuWrP9MDH5MBPbIqV92AaeXatLxBI9gBaebbnrfifHhDYfgasaacH8akY=wiFfYdH8Gipec8Eeeu0xXdbba9frFj0=OqFfea0dXdd9vqai=hGuQ8kuc9pgc9s8qqaq=dirpe0xb9q8qiLsFr0=vr0=vr0dc8meaabaqaciaacaGaaeqabaqabeGadaaakeaacqWGqbaudaWgaaWcbaGaemyqaeKaeiikaGIaemyAaKMaeiykaKIaeiilaWIaemiEaGNaeiikaGIaemyAaKMaeiykaKcabeaakiabg2da9maabmaabaWaaSGaaeaadaWcaaqaaiabd6eaonaaBaaaleaacqWGbbqqcqGGSaalcqWG4baEaeqaaaGcbaGaemOta40aaSbaaSqaaiabdgeabbqabaaaaaGcbaWaaSaaaeaacqWGobGtdaWgaaWcbaGaemiEaGhabeaaaOqaaiabd6eaonaaBaaaleaacqWG0baDcqWGVbWBcqWG0baDcqWGHbqycqWGSbaBaeqaaaaaaaaakiaawIcacaGLPaaaaaa@4C93@

Where *N*_*A*,*x *_is the number of amino acid type *A *with torsion angle combination *x*, *N*_*A *_the total number of amino acids of type *A*, *N*_*x *_the number of amino acids with torsion angle combination *x *and *N*_*total *_the total number of amino acids. The three terms *N*_*A*_, *N*_*x *_and *N*_*total *_can be derived from a background distribution of native structures, while *N*_*A*,*x *_is the observed state in the model being evaluated. Since the background distribution shows sharp transitions between highly populated and disallowed regions, no pseudo counts are used. Where needed, *N*_*x *_is set to a single count to avoid division by zero. A pseudo-energy scoring function *E *for a protein composed of *n *residues can be defined as:

E=∑i=1nEi=(∑i=1n−log⁡PA(i),x(i))
 MathType@MTEF@5@5@+=feaafiart1ev1aaatCvAUfKttLearuWrP9MDH5MBPbIqV92AaeXatLxBI9gBaebbnrfifHhDYfgasaacH8akY=wiFfYdH8Gipec8Eeeu0xXdbba9frFj0=OqFfea0dXdd9vqai=hGuQ8kuc9pgc9s8qqaq=dirpe0xb9q8qiLsFr0=vr0=vr0dc8meaabaqaciaacaGaaeqabaqabeGadaaakeaacqWGfbqrcqGH9aqpdaaeWbqaaiabdweafnaaBaaaleaacqWGPbqAaeqaaOGaeyypa0daleaacqWGPbqAcqGH9aqpcqaIXaqmaeaacqWGUbGBa0GaeyyeIuoakmaabmaabaWaaabCaeaacqGHsislcyGGSbaBcqGGVbWBcqGGNbWzcqWGqbaudaWgaaWcbaGaemyqaeKaeiikaGIaemyAaKMaeiykaKIaeiilaWIaemiEaGNaeiikaGIaemyAaKMaeiykaKcabeaaaeaacqWGPbqAcqGH9aqpcqaIXaqmaeaacqWGUbGBa0GaeyyeIuoaaOGaayjkaiaawMcaaaaa@51CF@

The torsion angle pseudo-energy score *E*_*i *_for the *i*-th residue *A *of a protein is thus a measure of the log propensity that amino acid type *A*(*i*) will have torsion angle combination *x*(*i*). *E*_*i *_< 0 indicates that *A*(*i*) is favoured relative to the mean of all 20 amino acids, whereas *E*_*i *_> 0 indicates that it is disfavoured. In order to be applied, both the background distribution and the relevant torsion angles have to be defined.

The Top500H database [42] was chosen to derive the background distribution in order to have a representative subset of high quality structures that is small enough to allow unbiased large-scale benchmarking of the PDB. It is a non-redundant, hand-picked set of 500 high-resolution X-ray crystallographic protein structures resolved to 1.8 Å or better resolution with no obvious errors and less than 60% sequence identity. In order to assess the effect of the background distribution quality, an alternative ensemble of 609 NMR structures (9,578 models) was also used. As the Ramachandran plot of NMR structures is largely determined by the force field used in refinement, this alternative ensemble contains blurred transitions and serves to highlight the effect of the background distribution on TAP score accuracy (see discussion).

Two free parameters, the choice of torsion angles to represent and the discretization of the data, have to be chosen in order to define the measured torsion angle combinations. The (ϕ,φ) angles were discretized as either 5, 10 or 20 degree bins. Since other torsion angles are less informative, but still important, a limited number of bins was used to represent the additional torsion angles [24]. Three bins were defined for the ω angle, distinguishing values [-180°, -150°], [+150°, +180°] and the rest. This was found to model the distribution of ω angles, where the *cis *(0°) state is very rare (except for proline) and the *trans *state preference is somewhat influenced by the Ramachandran plot [25] and has a slightly bimodal distribution around 180° (data not shown). Both the χ1 and χ2 torsion angles were discretized in eight bins centered on the canonical rotamer preferences. The total number of data points, i.e. non-terminal residues with all torsion angles available from the TOP500H is 100,245.

The torsion angle potential is a single body potential, representing the local structural preferences coded by each single residue in a polypeptide chain. Unlike other conventional statistical potentials, which are typically based on two-body interactions, it is therefore straightforward to calculate the global minimum *E*_>min _and maximum *E*_max _for a given protein sequence of length *n*:

Emin⁡=∑i=1nmin⁡(Ei)=∑i=1nmin⁡x(i)(−log⁡PA(i),x(i))
 MathType@MTEF@5@5@+=feaafiart1ev1aaatCvAUfKttLearuWrP9MDH5MBPbIqV92AaeXatLxBI9gBaebbnrfifHhDYfgasaacH8akY=wiFfYdH8Gipec8Eeeu0xXdbba9frFj0=OqFfea0dXdd9vqai=hGuQ8kuc9pgc9s8qqaq=dirpe0xb9q8qiLsFr0=vr0=vr0dc8meaabaqaciaacaGaaeqabaqabeGadaaakeaacqWGfbqrdaWgaaWcbaGagiyBa0MaeiyAaKMaeiOBa4gabeaakiabg2da9maaqahabaGagiyBa0MaeiyAaKMaeiOBa4MaeiikaGIaemyrau0aaSbaaSqaaiabdMgaPbqabaGccqGGPaqkcqGH9aqpaSqaaiabdMgaPjabg2da9iabigdaXaqaaiabd6gaUbqdcqGHris5aOWaaabCaeaadaWfqaqaaiGbc2gaTjabcMgaPjabc6gaUbWcbaGaemiEaGNaeiikaGIaemyAaKMaeiykaKcabeaakmaabmaabaGaeyOeI0IagiiBaWMaei4Ba8Maei4zaCMaemiuaa1aaSbaaSqaaiabdgeabjabcIcaOiabdMgaPjabcMcaPiabcYcaSiabdIha4jabcIcaOiabdMgaPjabcMcaPaqabaaakiaawIcacaGLPaaaaSqaaiabdMgaPjabg2da9iabigdaXaqaaiabd6gaUbqdcqGHris5aaaa@64F1@

Emax⁡=∑i=1nmax⁡(Ei)=∑i=1nmax⁡x(i)(−log⁡PA(i),x(i))
 MathType@MTEF@5@5@+=feaafiart1ev1aaatCvAUfKttLearuWrP9MDH5MBPbIqV92AaeXatLxBI9gBaebbnrfifHhDYfgasaacH8akY=wiFfYdH8Gipec8Eeeu0xXdbba9frFj0=OqFfea0dXdd9vqai=hGuQ8kuc9pgc9s8qqaq=dirpe0xb9q8qiLsFr0=vr0=vr0dc8meaabaqaciaacaGaaeqabaqabeGadaaakeaacqWGfbqrdaWgaaWcbaGagiyBa0MaeiyyaeMaeiiEaGhabeaakiabg2da9maaqahabaGagiyBa0MaeiyyaeMaeiiEaGNaeiikaGIaemyrau0aaSbaaSqaaiabdMgaPbqabaGccqGGPaqkcqGH9aqpaSqaaiabdMgaPjabg2da9iabigdaXaqaaiabd6gaUbqdcqGHris5aOWaaabCaeaadaWfqaqaaiGbc2gaTjabcggaHjabcIha4bWcbaGaemiEaGNaeiikaGIaemyAaKMaeiykaKcabeaakmaabmaabaGaeyOeI0IagiiBaWMaei4Ba8Maei4zaCMaemiuaa1aaSbaaSqaaiabdgeabjabcIcaOiabdMgaPjabcMcaPiabcYcaSiabdIha4jabcIcaOiabdMgaPjabcMcaPaqabaaakiaawIcacaGLPaaaaSqaaiabdMgaPjabg2da9iabigdaXaqaaiabd6gaUbqdcqGHris5aaaa@64FD@

where *E*_min _(*E*_max_) is the sum of the lowest (highest) pseudo-energy, i.e. highest (lowest) propensity, torsion angle combination for a residue of type *i*. Note that this definition makes no assumption about the physical plausibility of the overall conformation. Indeed, it is entirely possible that a sequence of minimal (or maximal) states would produce an impossibly "knotted" structure.

Given *E*_min _and *E*_max _of a protein sequence, it is possible to normalize the torsion angle potential score *E *as follows:

TAP=(E−Emin⁡)(Emax⁡−Emin⁡)
 MathType@MTEF@5@5@+=feaafiart1ev1aaatCvAUfKttLearuWrP9MDH5MBPbIqV92AaeXatLxBI9gBaebbnrfifHhDYfgasaacH8akY=wiFfYdH8Gipec8Eeeu0xXdbba9frFj0=OqFfea0dXdd9vqai=hGuQ8kuc9pgc9s8qqaq=dirpe0xb9q8qiLsFr0=vr0=vr0dc8meaabaqaciaacaGaaeqabaqabeGadaaakeaacqWGubavcqWGbbqqcqWGqbaucqGH9aqpdaWcaaqaaiabcIcaOiabdweafjabgkHiTiabdweafnaaBaaaleaacyGGTbqBcqGGPbqAcqGGUbGBaeqaaOGaeiykaKcabaGaeiikaGIaemyrau0aaSbaaSqaaiGbc2gaTjabcggaHjabcIha4bqabaGccqGHsislcqWGfbqrdaWgaaWcbaGagiyBa0MaeiyAaKMaeiOBa4gabeaakiabcMcaPaaaaaa@47BD@

The normalized torsion angle propensity TAP gives a rough indication of the degree of "nativeness" of a protein model. The value will be close to 1 for the native structure and close to 0 for structures with largely incompatible sequences. It is therefore a measure of compatibility between sequence and structure.

### Data sets

In order to evaluate the method for structure quality estimation on a large set, we downloaded the ASTRAL database [43] version 1.69 (December 2005) containing 68,057 domain sequences for 24,978 PDB structures [1] in 2,844 SCOP families [44]. Of these, we considered only 17,685 structures determined by X-ray crystallography. To avoid bias towards the background distribution used to derive the torsion angle potential, we remove 355 structures belonging to the same SCOP families as the TOP500H structures. The programs failed to load ca. 1–2% of the structures, containing anomalous data usually from very old PDB files. The all PDB set is composed of 13,691 structures with both valid resolution and R-free values and results for all tested methods (see below). For protein complexes in this set, the scores are calculated for every chain and averaged. Luzzati and σ_A _values were only used when derived from cross-validated data, in analogy to R-free. The DPI values were calculated from the PDB structures according to the published formula [9]. Table 1 summarizes the available structures.

The second data set is based on obsolete PDB entries. A list containing pairs of PDB codes of PDB entries rendered obsolete since January 1990 and their replacement was downloaded from the PDB site. This resulted in a set of 494 pairs of PDB codes for which all methods tested produced valid output. The details for both data sets are available as supplementary material.

### Methods used for comparison

The comparison with published methods is based on PROCHECK [19], WHAT_CHECK [16], HOPPscore [22] and FRST [23]. All three programs were either downloaded from the author's website or directly requested (HOPPscore). For PROCHECK, the overall G-factor was used. WHAT_CHECK analysis is based on nine overall quality indicators available from the Pdbfinder2 database [45]. These are: overall quality (WC_Qual) expressed as a sum of various terms, torsion angles (WC_Tors), Ramachandran plot appearance (WC_Rama), chirality (WC_Chir), backbone conformation (WC_Back), rotamer normality (WC_Rot), χ-1/χ-2 rotamer normality (WC_Chi1&2) and 1^st ^and 2^nd ^generation packing quality (WC_pack1 and WC_pack2). It should be noted that WC_Pack1 and WC_Pack2 are measures based on contact analysis. For HOPPscore, the five (Hopp-5) through single residue (Hopp-1) scores were calculated with default parameters. FRST is a linear combination of four different statistical potentials [23]: a pairwise potential (RAPDF), solvation potential (SOLV), a simplified count of main chain hydrogen bonds (HYDB) and the torsion angle potential (TORS) on which TAP is built. All five (partial) potentials were used for analysis. The PROCHECK, WC_Rama, Hopp1 and TORS scores essentially represent a quantification of the structural fit with the Ramachandran plot.

### Evaluation criteria

Analysis of the results is based on several criteria. For the obsolete PDB set, let *S*_*new *_and *S*_*old *_denote the score of the new resp. old structure. The first criterion is the number of pairs in which the scoring function correctly recognizes the improved structure, i.e. S_*new *_> S_*old*_. The separation Z-score *z *is defined as:

z=(Snew−Sold)σold
 MathType@MTEF@5@5@+=feaafiart1ev1aaatCvAUfKttLearuWrP9MDH5MBPbIqV92AaeXatLxBI9gBaebbnrfifHhDYfgasaacH8akY=wiFfYdH8Gipec8Eeeu0xXdbba9frFj0=OqFfea0dXdd9vqai=hGuQ8kuc9pgc9s8qqaq=dirpe0xb9q8qiLsFr0=vr0=vr0dc8meaabaqaciaacaGaaeqabaqabeGadaaakeaacqWG6bGEcqGH9aqpdaWcaaqaaiabcIcaOiabdofatnaaBaaaleaacqWGUbGBcqWGLbqzcqWG3bWDaeqaaOGaeyOeI0Iaem4uam1aaSbaaSqaaiabd+gaVjabdYgaSjabdsgaKbqabaGccqGGPaqkaeaaiiGacqWFdpWCdaWgaaWcbaGaem4Ba8MaemiBaWMaemizaqgabeaaaaaaaa@42FF@

where σ_*old *_is the standard deviation calculated over all S_*old *_for that particular method.

The experimental and computational parameters are analyzed in terms of Pearson correlation coefficient *cc *over the all PDB set. Fraction enrichment *FE *measures the percentage of good structures recognized at a threshold level *t *by each method [23]. The structures are first ranked by R-free and by each method. *FE *at threshold *t *measures the percentage of structures in common between the top *x *percent of both lists. For the present work, the *FE *threshold is plotted in discrete steps of 5% from 5% to 50%. Intuitively, it becomes progressively easier for methods to have higher *FE *values at higher threshold levels. E.g. a good, but not perfect, method will be able to detect most of the good structures at *t *= 50%, but mostly fail at *t *= 5%.

Last but not least, confidence estimates were derived for TAP using accuracy (*acc*) and coverage (*cov*):

acc=TP(TP+FN)
 MathType@MTEF@5@5@+=feaafiart1ev1aaatCvAUfKttLearuWrP9MDH5MBPbIqV92AaeXatLxBI9gBaebbnrfifHhDYfgasaacH8akY=wiFfYdH8Gipec8Eeeu0xXdbba9frFj0=OqFfea0dXdd9vqai=hGuQ8kuc9pgc9s8qqaq=dirpe0xb9q8qiLsFr0=vr0=vr0dc8meaabaqaciaacaGaaeqabaqabeGadaaakeaacqWGHbqycqWGJbWycqWGJbWycqGH9aqpdaWcaaqaaiabdsfaujabdcfaqbqaaiabcIcaOiabdsfaujabdcfaqjabgUcaRiabdAeagjabd6eaojabcMcaPaaaaaa@3B2D@

cov⁡=TP(TP+FP)
 MathType@MTEF@5@5@+=feaafiart1ev1aaatCvAUfKttLearuWrP9MDH5MBPbIqV92AaeXatLxBI9gBaebbnrfifHhDYfgasaacH8akY=wiFfYdH8Gipec8Eeeu0xXdbba9frFj0=OqFfea0dXdd9vqai=hGuQ8kuc9pgc9s8qqaq=dirpe0xb9q8qiLsFr0=vr0=vr0dc8meaabaqaciaacaGaaeqabaqabeGadaaakeaacyGGJbWycqGGVbWBcqGG2bGDcqGH9aqpdaWcaaqaaiabdsfaujabdcfaqbqaaiabcIcaOiabdsfaujabdcfaqjabgUcaRiabdAeagjabdcfaqjabcMcaPaaaaaa@3B72@

where *TP *are the true positive predictions, i.e. where TAP correctly predicts a structure to be of a given quality class. (*TP+FN*) are all predictions made by TAP and (*TP+FP*) are all structures having a given quality class.

## Availability and requirements

The TAP software is freely accessibile as a web server at . An executable version, written in ANSI C++ and precompiled for Linux machines, is also freely available for academic usage from . Please contact the author for obtaining the source code and/or for commercial usage.

## Authors' contributions

SCET designed the study, performed the experiments, analyzed the results and wrote the paper. RB contributed the X-ray crystallographic background and to the analysis of the data. Both authors have read and approved the final manuscript.

**Figure 5:**
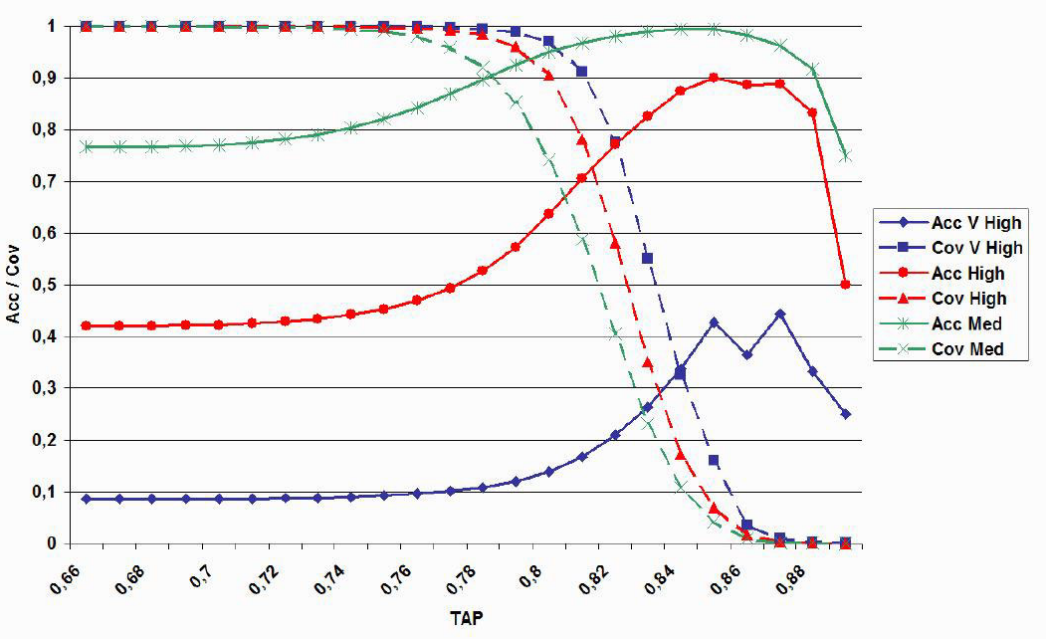
**Accuracy and coverage plot for TAP score on the all PDB set**. Accuracy (acc) and coverage (cov) are plotted for three experimental quality classes (medium, high, very high) as a function of TAP score threshold.

## Supplementary Material

Additional file 1 **All PDB dataset**. This text file contains the PDB code, R-free, resolution and TAP score values, one protein per line, for all proteins in the all PDB dataset. Click here for file

Additional file 2 **Obsolete PDB dataset**. This text file contains the year rendered obsolete, obsolete PDB code, TAP score of the obsolete structure, replacement PDB code and TAP score of the replacement structure, one protein per line, for all proteins in the obsolete PDB dataset. Click here for file
